# Terminology - clarifying the current confusion and presenting the correct terminology

**DOI:** 10.3389/fopht.2023.1249557

**Published:** 2023-11-14

**Authors:** Ejaz Ansari

**Affiliations:** ^1^ Ophthalmology, Maidstone & Tunbridge Wells NHS Trust, Maidstone, United Kingdom; ^2^ Institute of Medical Sciences, Christ Church Canterbury University, Canterbury, United Kingdom

**Keywords:** presbyopia-correcting, EDoF lenses, increased range of focus, dysphotopsias, depth of field

## Abstract

Most readers will be familiar with terminology (from meetings and conferences) that we, the editors, would deem to be incorrect or confusing. In general, we all tend to accept the information conveyed to us without really questioning what the terms truly mean. Therefore, the subsequent chapters discussing the IOLs in question should be studied with this in mind since a lot of familiar terminology will be used in those chapters, which, strictly speaking, may not be in line with the most accurate terminology and classification. The reader is encouraged to read this full article to be mindful of the current confusion surrounding IOL terminology and what the correct terminology should be. This is so that one is better prepared to understand and question the mechanism of action and side effects of IOLs that one is planning to use.

## Introduction

When describing intraocular lenses (IOLs) that are used for the management of presbyopia, certain terms are repeated and become embedded, often without question or further enquiry by the listener. Phrases such as “premium IOL, EDoF (extended depth of focus), and “fewer dyspohotopsias” are well known by the cataract surgery fraternity. But what do these phrases mean and is it correct to use them?

## Discussion

A premium is the cost of insurance, that is usually an amount paid annually. “At a premium” means scarce and in demand, or above the usual or nominal price, the latter meaning being most appropriate in this case. However, it does not indicate how the IOL functions for the patient’s benefit. Another phrase that is used interchangeably with premium is “presbyopia-correcting”. This is misleading since these IOLs do not *correct* presbyopia, but rather increase the range of focus (RoF). High- performance IOL (HPIOL) is a better descriptor.

What does EDoF mean? The acronym EDoF comes from photography, where it refers to the *extended depth of field* achieved by reducing either the aperture or the magnification of an optical system. EDoF is used liberally by manufacturers and surgeons who promote enhanced vision quality and range of focus compared to multifocal IOLs and monofocal IOLs respectively.

In actual fact, the only lenses that truly extend the depth of field are small-aperture (i.e., pinhole) lenses, e.g., the IC8 (AcuFocus) and XtraFocus Pinhole Implant (Morcher). The remainder are either extended range of focus (EROF) or increased range of focus (IROF) implants. Full range of focus (FROF) would apply to trifocals.

The American National Standards Institute (ANSI) (Z80.35-2018) standard for EDoF IOLs require implants to have intermediate vision (67 cm) of 20/30 in at least 50% of recipients. There is no mention of dyspohotopsias, but clinicians tend to associate EDoF IOLs with fewer dyspohotopsias! This misconception that EDoF equals fewer or no dyspohotopsias has stoked the flames of enthusiasm in many IOL manufacturers.

In actual fact, the mechanisms of action of some EDoF IOLs may result in halos, glare, starbursts, and unsharp focus. For example, the mechanism utilised to increase the range of focus may not follow the principle of elongating the conoid of Sturm. Instead, refractive, diffractive or a combination of mechanisms are used resulting in dyspohotopsias that are associated with bifocal and trifocal IOLs anyway. This can lead to dissatisfied patients who have been informed erroneously that they will not experience dyspohotopsias with an EDoF IOL.

An alternative classification of this category of IOLs has been suggested by the American European Congress of Ophthalmic Surgery (AECOS) (1). A committee of four surgeons set about to demystify the myth that EDoF IOLs leading to fewer dyspohotopsias and the confusion arising from the variety of IOLs in this category (**Table 1**). They subdivided the concept of lens performance into three main components:

1. Range of focus
2. Mechanisms of action
3. Dyspohotopsias (1)

**Table 1:** AECOS classification of IOLs: terminology separates lens performance (i.e., ROF) from mechanism of action.

CATEGORY 1
Range of focus (ROF)
• Single focus (i.e., monofocal- spheric or aspheric)
• Increased ROF (IROF) NB: *increased* is preferable to extended to avoid confusion with extended depth of focus.
• Full ROF (FROF)
CATEGORY 2
Mechanism of action
• Accommodative
• Small aperture
• Diffractive
• Zonal refractive
• Other
• Combined mechanism
Dyspohotopsias (N.B. incidence and type of postoperative dyspohotopsias for each type of lens was not included in AECOS’ proposed categorization of IOLs)
• Glare
• Halos
• Starbursts
• Other

(Reprinted with permission of Sheraz M. Daya, MD, FACP, FACS, FRCSEd, FRCOphth, and Bryn Mawr Communications. Ambati B, Daya SM, Trinh T. Standardizing terms in cataract and refractive surgery. Cataract & Refractive Surgery Today Europe. March/April 2023;18(2):50-51) (1).

It is true that other variables such as neuroadaptation, expectations, corneal optics, post-operative pupil size and axial length have a part to play in final lens performance, however these were not included in the classification to avoid too much complexity.

Another consideration when selecting IROF IOLs is their dependence on good centration along the visual axis. This is described in the fourth column of the **Table 2**.

**Table 2:** Increased range of focus IOLs (2).

COMPANY	IOL	MECHANISM	ZONE OF MODIFICATION; DEPENDENCE ON VISUAL AXIS
Acufocus	IC8	Single aperture/EDoF	Central; high dependence
Alcon	Vivity	Zonal refractive + spherical aberration	Paracentral; low dependence
Bausch & Lomb	Luxsmart	Zonal refractive + spherical aberration	
BVI/Physiol	Isopure	Other; spherical aberration	Central; high dependence
Cristalens	Reverso (Piggy Back)	Refractive bifocal	
J&J Vision	Symfony	Diffractive +	
	Eyhance	Zonal refractive +	Central; high dependence
Oculentis	Comfort	Zonal refractive	Central; high dependence
Swiss Advanced Vision	Lucidis	Zonal refractive	
Zeiss	AT Lara	Diffractive	
Cutting Edge SAS	Synthesis Plus	Zonal refractive	Paracentral; low dependence

The + suffix refers to more than one mechanism of action (3, 4).

## Conclusion

It is important to categorize IOLs in clear and simple language for the benefit of patients and surgeons alike. Obviously, clear, and simple language assists the surgeon in making sense of all the variety of options available and what would suit individual patients. Similarly, it helps patients to be more well informed of the IOL that suits their needs. Collaboration between stakeholders is essential to produce terminology that is clearer to everyone.

*Falsehood flies, and the truth comes limping after it; so that when men become undeceived, it’s too late; the jest is over, and the tale has had its effect.* Jonathan Swift 1710

## Data availability statement

The original contributions presented in the study are included in the article/supplementary material. Further inquiries can be directed to the corresponding author.

## Author contributions

The author confirms being the sole contributor of this work and has approved it for publication.
